# Electromagnetic Exposure from RF Antennas on Subway Station Attendant: A Thermal Analysis

**DOI:** 10.3390/s26020709

**Published:** 2026-01-21

**Authors:** Jin Li, Qianqian Zhang, Mai Lu

**Affiliations:** Key Laboratory of Opto-Electronic Technology and Intelligent Control of the Ministry of Education, Lanzhou Jiaotong University, Lanzhou 730070, China; 12242106@stu.lzjtu.edu.cn (Q.Z.); mai.lu@hotmail.com (M.L.)

**Keywords:** subway station, station attendant, RF antenna, occupational electromagnetic exposure, temperature rise

## Abstract

With the rapid development of wireless communication systems, the electromagnetic environment in subway stations has become increasingly complex, raising concerns about the long-term safety of station attendants who are chronically exposed to radiofrequency (RF) fields. At present, multiphysics analyses specifically addressing RF antenna exposure scenarios for subway attendants remain limited. To assess occupational electromagnetic exposure risks, this paper establishes a comprehensive electromagnetic–thermal coupling simulation model incorporating RF antennas, station-platform structures, and a realistic human model with organs including the brain, heart, and liver. Using the finite-element software COMSOL Multiphysics (v.6.3), numerical simulations are performed to calculate the specific absorption rate (SAR) in the trunk and major organs of the subway station attendant at RF antennas frequencies of 900 MHz, 2600 MHz, and 3500 MHz, as well as the temperature rise distribution of the human trunk and important tissues and organs under different initial temperatures of the environment. The results show that among the three frequencies, the maximum SAR of 5.55 × $10^{-4}$ W/kg occurs in the trunk at 3500 MHz. Tissue temperatures reach thermal steady state after 30 min of exposure, with the maximum temperature rises occurring in the brain at an ambient temperature of 18 °C and an operating frequency of 900 MHz, reaching 0.2123 °C. Across all simulated scenarios, both SAR values and temperature rises remain significantly below the occupational exposure limits established by the International Commission on Non-Ionizing Radiation Protection (ICNIRP). These findings indicate that RF radiation generated by antennas in the subway station environment poses low health risks to female station attendants of similar physical characteristics to the Ella model. This study provides a scientific reference for the occupational RF protection of subway personnel and contributes data for the development of electromagnetic exposure standards in rail transit systems.

## 1. Introduction

According to data released by the International Association of Public Transport (UITP) in May 2025, the total length of global subway networks exceeded 20,000 km by the end of 2023, with China accounting for 43% of this cumulative mileage [1]. In August 2025, China’s urban rail transit system recorded a passenger volume of 2.88 billion trips and a station entry volume of 1.71 billion trips, with subway and light rail systems accounting for 1.65 billion of the entries [2]. Over the past decade, China’s subway has entered a period of rapid development, and with continuous increases in line length and passenger volume, it has become the world’s largest subway construction market. With the emergence of smart subway infrastructure, the subway communication system has been rapidly upgraded, achieving comprehensive 5G coverage across stations and key transport hubs [3]. Subway stations are characterized by high passenger density and substantial data traffic demand, with radio frequency (RF) sources operating across distinct frequency bands and supporting diverse communication functions; the various frequencies coexist within subway stations, resulting in complex RF electromagnetic environments [4,5,6].

**Figure 1:**
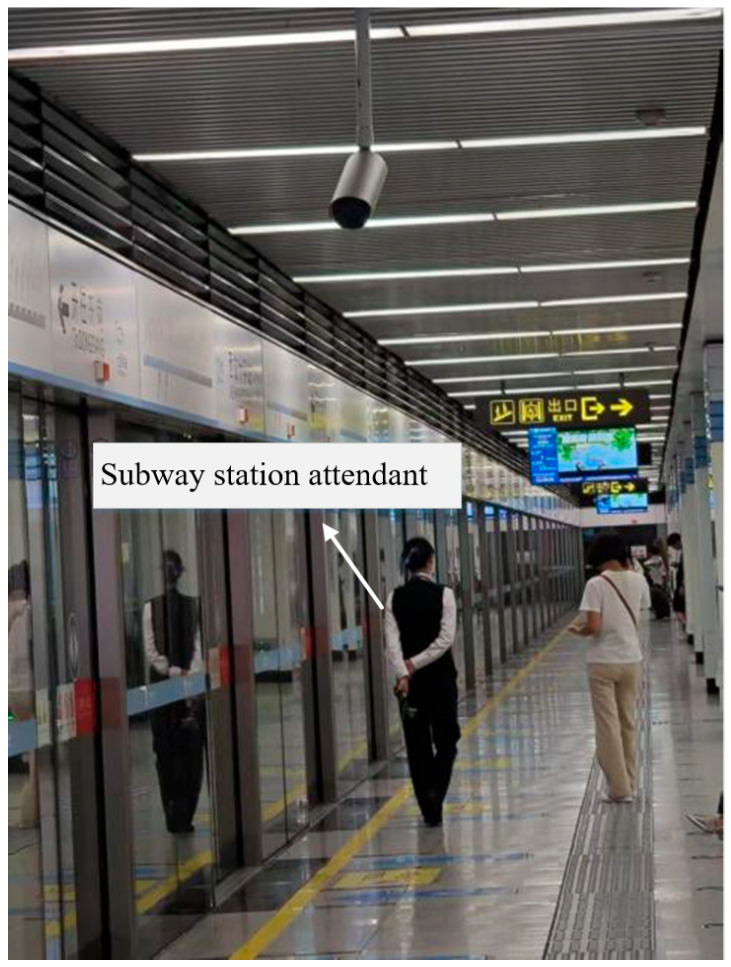
Subway attendant working on the platform.

As the radiating terminals of subway wireless communication systems, RF antennas are typically mounted on the ceilings of station platforms [7]. Subway station attendants, whose responsibilities include managing passenger boarding and alighting, coordinating platform operations, and guiding passenger flow, are consequently exposed to the complex electromagnetic environment generated by these RF antennas over extended periods of time. Figure 1 shows a subway attendant working on the platform.

The growing demand for communication has led to a complex occupational electromagnetic environment, and the potential adverse health effects associated with RF exposure have drawn widespread attention. The International Commission on Non-Ionizing Radiation Protection (ICNIRP) and the Institute of Electrical and Electronics Engineers (IEEE) have, respectively, established and updated guidelines for limiting electromagnetic field exposure to ensure protection for the occupational personnel and the general public against RF overexposure [8,9]. At present, there have been numerous studies on the safety of human electromagnetic exposure caused by RF antennas in different environments [10,11,12]. According to the published literature, the short-term health effects of RF electromagnetic exposure include headaches, chest distress, nausea, sleep disorders, and neurasthenia [13,14]. The long-term effects include possible cancer, brain tumors, and DNA damage [15,16]. Consequently, the safety of occupational electromagnetic exposure among personnel in rail transit radio frequency environments merits careful attention.

When the human body is exposed to RF electromagnetic fields, the absorbed electromagnetic energy is converted into heat, leading to an increase in tissue temperature, and the excessive temperature rise may induce adverse physiological responses [17]. In recent years, numerous studies have been conducted on the rise in human tissue temperature associated with electromagnetic exposure [18,19,20]. Shirun Wang et al. integrated a monopole array antenna into a shark-fin antenna on a car roof for Vehicle-to-Vehicle (V2V) communications and evaluated the specific absorption rate (SAR) and temperature rise in the human body in a smart mobility communication scenario operating at 5.9 GHz [18]. Bhargava et al. studied specific absorption rate and temperature rise in the human head due to overexposure to mobile phone radiation with different usage patterns [19]. Ugljesa Z et al. evaluated the SAR and thermal rise in the brains of adult males exposed to mobile phone radiation, reporting that continuous exposure at 900 MHz for one hour may lead to an increase in brain temperature of approximately 0.09 °C [20]. Collectively, these studies highlight the increasing scientific attention to electromagnetic–thermal coupling phenomena driven by advancements in wireless communication technologies. However, systematic research on the multiphysical field coupling problem concerning subway station attendants under RF radiation remains lacking.

Currently, the existing research on electromagnetic radiation in rail transit systems has primarily focused on wireless signal coverage, electromagnetic interference mitigation, and environmental field strength measurements [21,22,23,24]. WenYing Zhou et al. proposed a dual-band trackside antenna based on meta-materials to enable 5G communication in tunnel environments and assessed its electromagnetic exposure impact on personnel working in such settings [21]. Yixuan Wang et al. employed an integrated approach combining on-vehicle mobile measurements and fixed-point measurements to conduct a comprehensive and systematic assessment of electromagnetic exposure levels along two railway lines in Lanzhou, China, across distinct time intervals [22]. Ashraf et al. investigated RF electromagnetic exposure in underground environments, such as subways, tunnels, and mines, to assess the exposure levels associated with communication devices in these settings [23]. Xuejian Zhang et al. conducted numerous narrowband channel measurements in realistic subway tunnels and obtained sufficient data, with a focus on investigating the wireless propagation characteristics of 1.8 and 5.8 GHz in four tunnel scenarios [24]. Studies addressing occupational RF exposure among rail transit workers remain limited. Nadine Worel et al. examined the effects of high-frequency and extremely low-frequency electromagnetic fields on DNA integrity under occupational exposure conditions, reporting no significant DNA alterations [25]. Jin Li et al. evaluated the safety of occupational electromagnetic exposure associated with leaky coaxial cables and its effects on subway attendants [26]. Maxime Turuban et al. analyzed RF exposure levels among various occupational groups in Spain and France, finding generally low exposure [27]. Given that subway station attendants are subject to prolonged exposure to electromagnetic fields generated by RF antennas, there is an urgent need for a quantitative assessment of temperature rise in human tissues to enable the evaluation of occupational RF exposure risks.

Therefore, in this paper, a high-fidelity anatomical model of a subway station attendant, including key internal organs and 5G-band antenna models are designed based on the COMSOL Multiphysics simulation platform. Numerical simulations were conducted to evaluate the SAR and temperature rise in the human body and critical tissues of the subway station attendant during routine work scenarios. The results were compared with the exposure limits specified by the ICNIRP to evaluate the occupational RF exposure safety of the subway station attendant. These findings provide scientific support for the development of electromagnetic exposure protection strategies and standards in rail transit systems.

## 2. Models and Methods

### 2.1. RF Antenna Model

**Figure 2:**
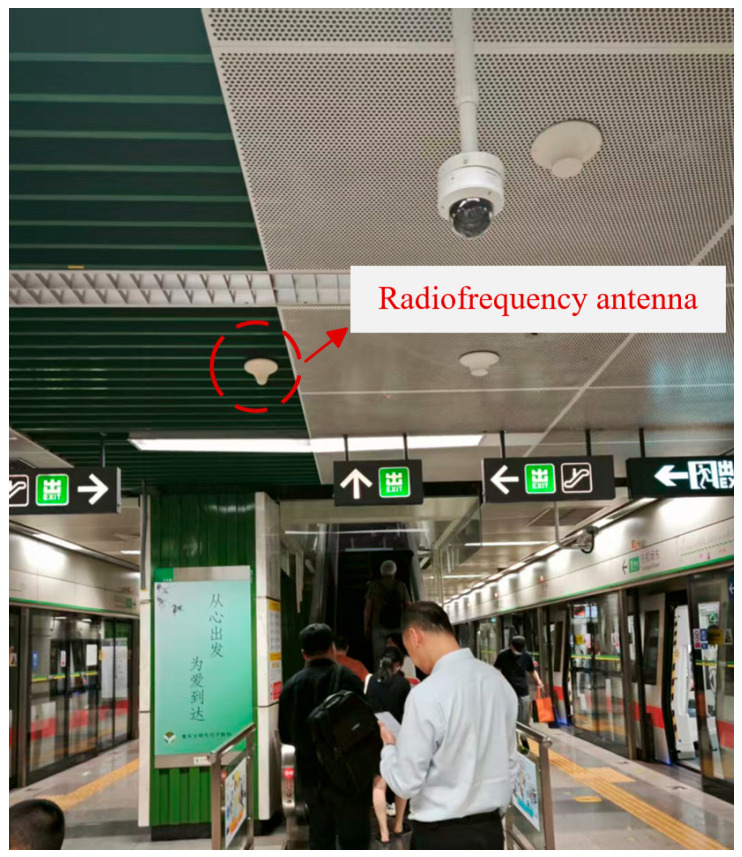
RF antenna in a subway station.

**Figure 3:**
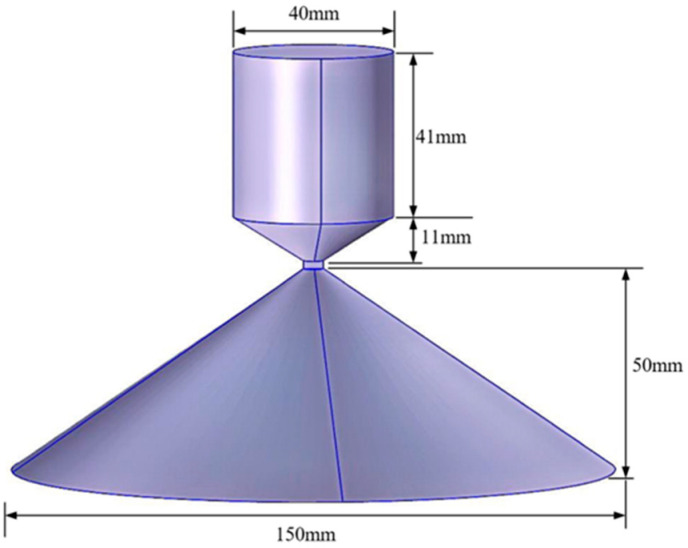
Structure of RF antenna model.

Subway stations constitute a distinctive indoor environment characterized by high passenger density and a substantial demand for mobile communication services. RF antennas, as typical radiation sources of wireless communication systems in the subway station, deliver comprehensive electromagnetic field strength coverage across the working zones of a subway station attendant. These antennas are typically installed beneath the station ceiling, as shown in Figure 2. In this paper, a radio frequency antenna covering the 5G frequency band is designed. The antenna is a variation in the double-cone structure; its upper cone is composed of a cylinder with a diameter of 40 mm and a height of 41 mm, and an inverted cone with a height of 11 mm. The lower cone is a conical structure with a bottom diameter of 150 mm and a height of 50 mm; a lumped port is arranged between the two cones. The antenna’s operating frequency bands are 900 MHz, 2600 MHz, and 3500 MHz. Both the upper and lower cones of the antenna are made of aluminum, fed by a 50 Ω lumped port, and the antenna power is set to 50 mW, as shown in Figure 3.

**Figure 4:**
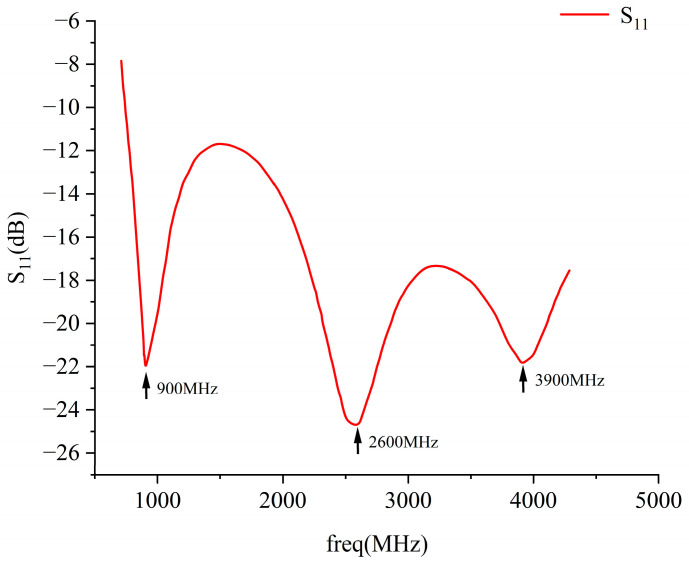
Antenna S_11_ parameter.

**Figure 5:**
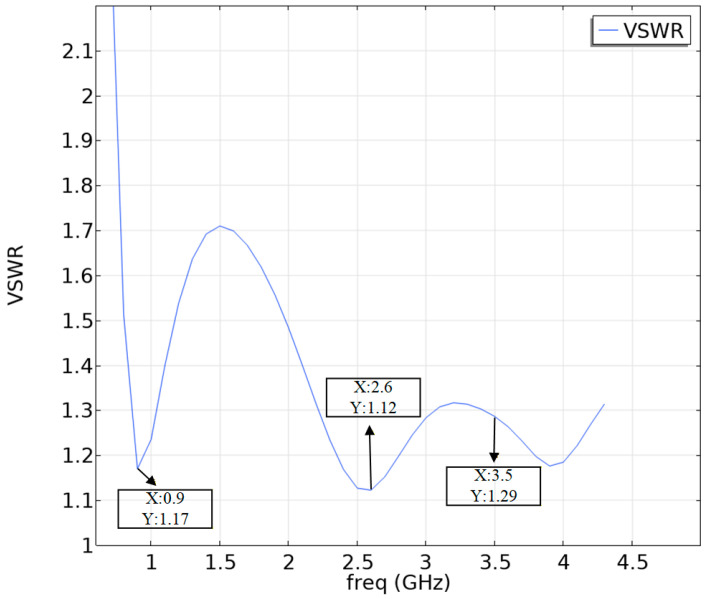
VSWR.

**Figure 6:**
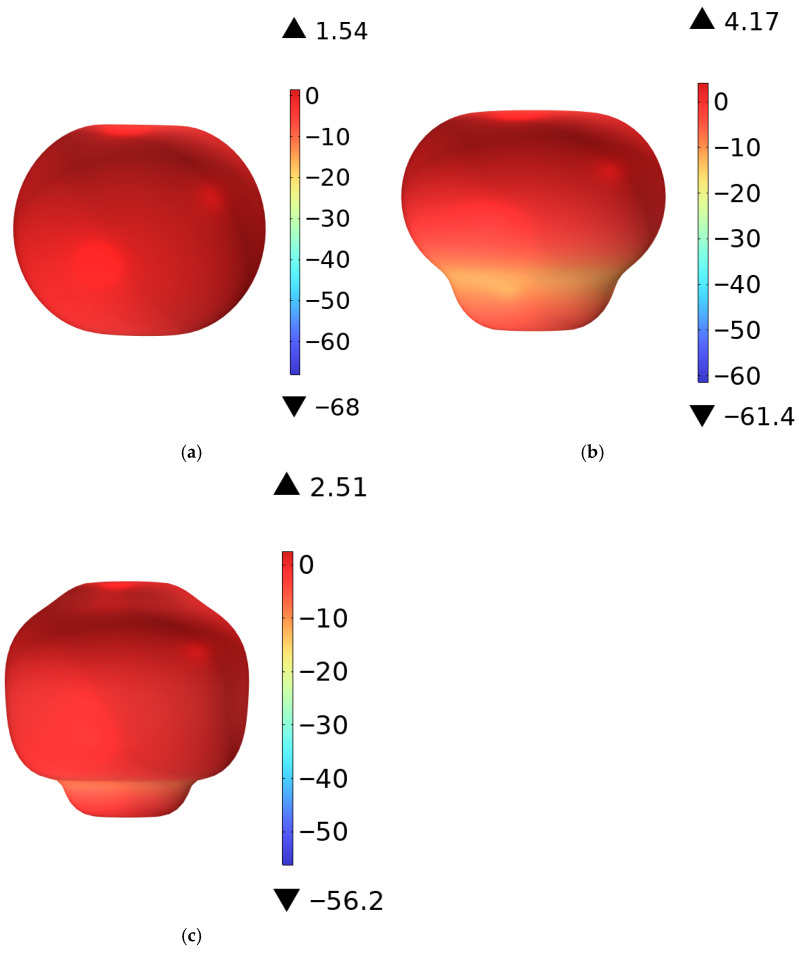
Gain of the RF antenna: (**a**) 900 MHz, (**b**) 2600 MHz, and (**c**) 3500 MHz.

**Figure 7:**
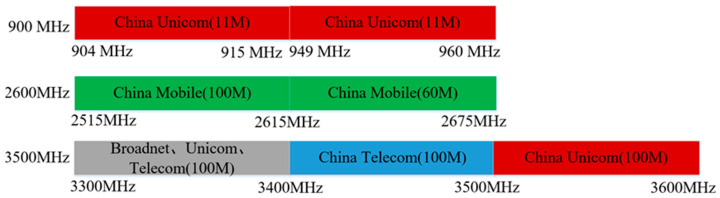
The 5G frequency band of the civil communication system in the subway station.

Using the multiphysics coupling software COMSOL for simulation, the results show that the $S_{11}$ parameter of this RF antenna is below −10 dB in the civilian communication system frequency bands of 900 MHz, 2600 MHz, and 3500 MHz [28,29]. The $S_{11}$ parameter, namely the reflection coefficient, represents the ratio of reflected incident electromagnetic waves, and its variation curve is shown in Figure 4. The voltage standing wave ratio (VSWR) is a key parameter for evaluating the matching efficiency of the antenna system. Its curve is shown in Figure 5, which is less than 1.5 at 900 MHz, and less than 2.0 at 2600 MHz and 3500 MHz. The three-dimensional far-field radiation pattern of the antenna is shown in Figure 6a–c. The gain is 1.54 dBi at 900 MHz, greater than 1.5; the gains at 2600 MHz and 3500 MHz are 4.17 dBi and 2.51 dBi, both greater than 2.5. As an ultra-wideband antenna, it meets the design requirements, covering the 2600 MHz and 3500 MHz frequencies of a 5G network. The 5G frequency bands of the civil communication system in the subway station are shown in Figure 7.

### 2.2. Numerical Human Body Model

In 1941, Kenneth S. Cole and Robert H. Cole developed a method for modeling the dielectric behavior of dielectric materials, known as the Cole–Cole model [30], which describes the dispersion and absorption characteristics of dielectrics over a broad frequency range, and shows that conductivity and relative permittivity depend on frequency. Building upon this foundational work, Gabriel et al. experimentally measured the relative permittivity and conductivity of 17 biological tissues from 10 Hz to 20 GHz, and subsequently developed the four-pole Cole–Cole model in 1996 [31,32,33]. The four-pole Cole–Cole formulation is shown in Equation (1).

(1) $$\varepsilon (\omega )=\varepsilon^{\prime }-j\varepsilon^{\prime \prime }=\varepsilon_{\infty }+\sum_{n=1}^{4}\frac{∆\varepsilon_{n}}{1+{\left(j\omega \tau_{n}\right)}^{1-\alpha_{n}}}+\frac{\sigma_{i}}{j\omega \varepsilon_{0}}$$

In Equation (1), *ε*(*ω*) denotes the complex relative permittivity, with units of F/m, describing the dielectric response of a material to an applied electric field; ε′ represents the real part of the complex relative permittivity, and ε″ represents the imaginary part. $\varepsilon_{\infty }$ denotes the relative permittivity at infinite frequency; $∆\varepsilon_{n}$ is the dielectric increment in the n-th dispersion region relative to the static relative permittivity; $\varepsilon_{0}\;$= 8.854187817 × 10^−12^ F/m represents the permittivity of free space; $\sigma_{i}$ is the static electrical conductivity with units of S/m; $\tau_{n}$ signifies the relaxation time constant associated with the n-th dispersion process; and *ω* = 2*π*f is the angular frequency.

**Figure 8:**
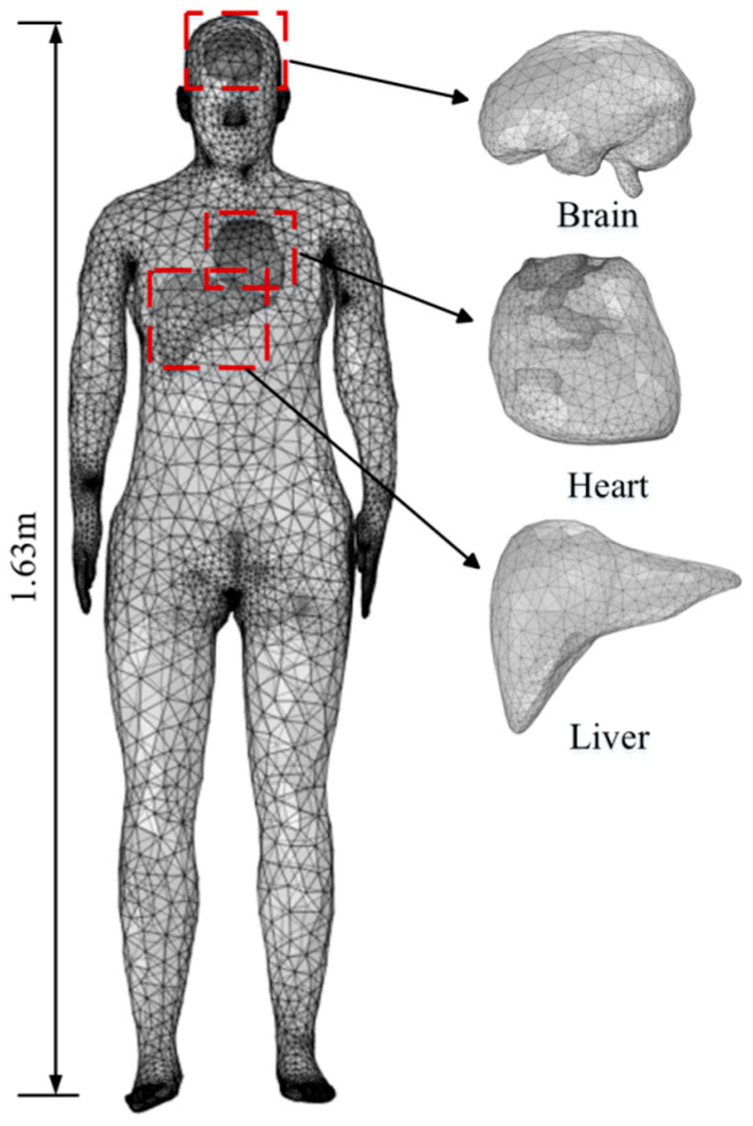
Human model of the subway attendant.

The human body model used in this study is obtained from the Virtual Family Project and consists of a high-resolution, MRI-based anatomical model of an adult female, Ella [34], as shown in Figure 8. The model has a height of 1.63 m and includes major internal organs such as the brain, heart, and liver. For the brain, the electrical conductivity and permittivity values are derived by computing the arithmetic average of the corresponding properties of white matter and gray matter. The dielectric parameters of the trunk are taken as the unweighted arithmetic average of the dielectric parameters of four tissues: skin, bone, muscle, and blood. The relative permittivity and electrical conductivity of each tissue are calculated at frequencies of 900 MHz, 2600 MHz, and 3500 MHz using the four-pole Cole–Cole model [35], with the results shown in Table 1.

Table 2 presents the thermal properties of the trunk, brain, and heart tissues employed in the human body model [19,36,37], which are essential for the computation of temperature rise in biological tissues. Where *ρ* is the tissue density, *k* denotes thermal conductivity, *C* signifies specific heat capacity, and $\omega_{b}$ refers to the blood perfusion rate.

### 2.3. Electromagnetic Exposure Scenario Model

**Figure 9:**
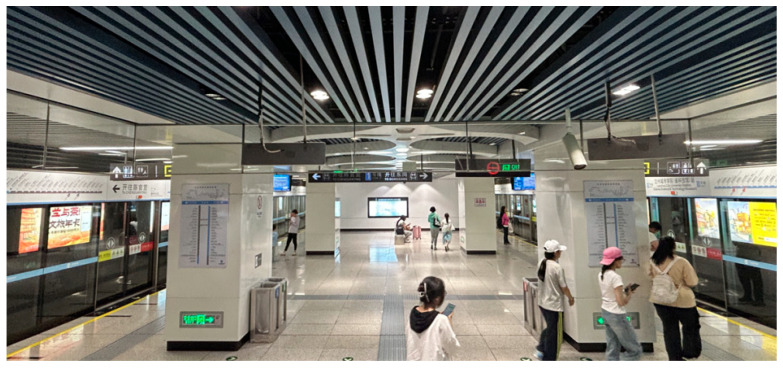
Working scenario of the subway station attendant.

**Figure 10:**
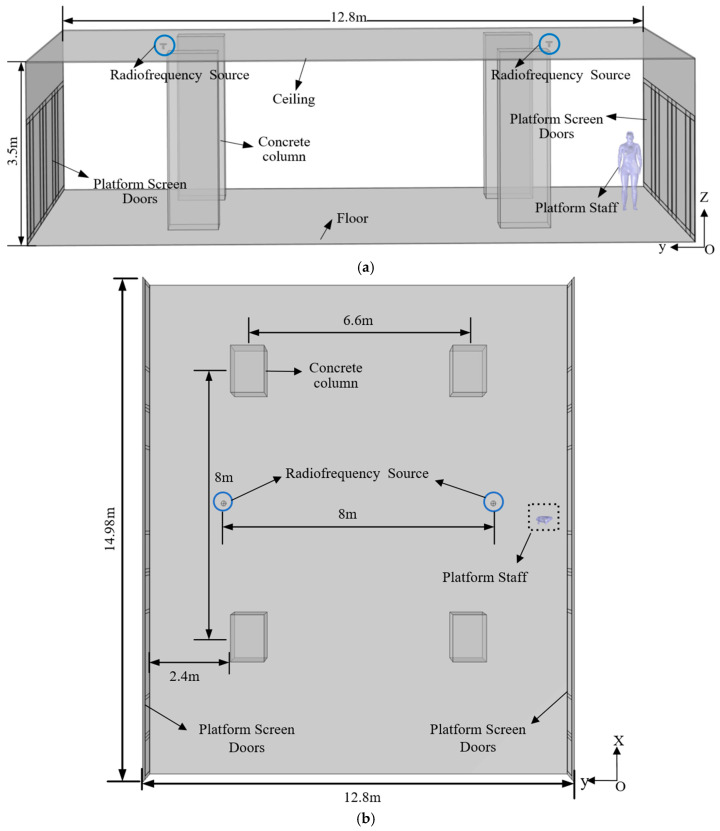
Electromagnetic exposure scenario model: (**a**) YZ plane, (**b**) XY plane.

Figure 9 shows a typical working scenario of a subway station attendant. Platform screen doors (PSDs) are installed on both sides of the platform. The platform features four concrete columns, and RF antennas are mounted beneath the ceiling, with one antenna positioned above each track direction. Using COMSOL software, the electromagnetic exposure scenario model employed in this study was developed, as shown in Figure 10a,b. Figure 10a presents the YZ-plane view, while Figure 10b displays the XY-plane view. As illustrated in Figure 10, the vertical distance between the ceiling and the platform floor is 3.5 m; the horizontal spacing between the two RF antennas is 8 m; and each antenna is located 2.4 m from the adjacent platform screen door. The PSDs have a height of 2.4 m and consist of fixed doors measuring 2480 mm × 2200 mm and sliding doors measuring 2080 mm × 2200 mm. An aluminum-alloy shielding panel—1.1 m in height—is installed above the PSD structure. The distance between the PSDs on opposite sides of the platform is 12.8 m. Each concrete column has dimensions of 1400 mm × 1000 mm × 3500 mm. During operation, the station attendant stands at a distance of 0.5 m from the PSD.

**Figure 11:**
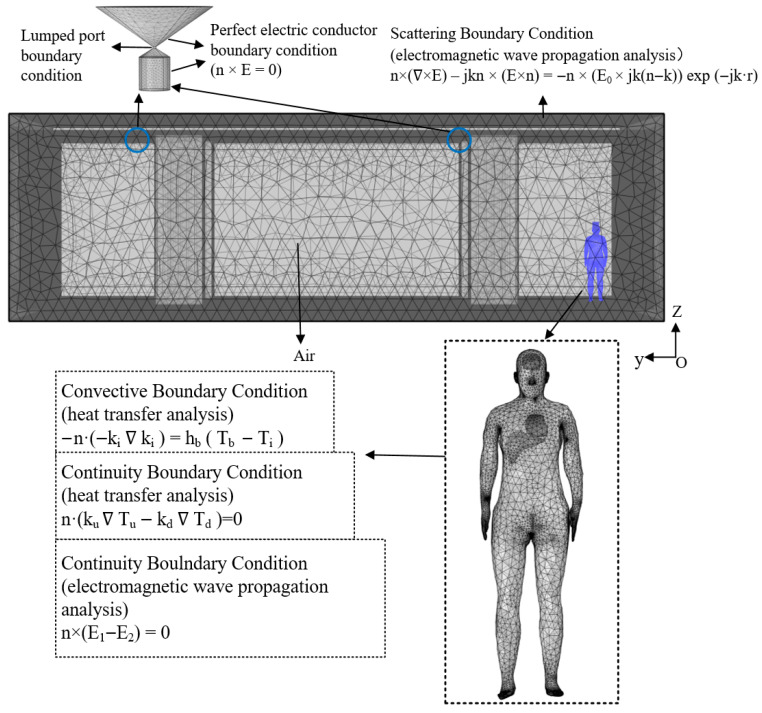
Meshing of the model and setting of boundary conditions.

A rectangular air domain measuring 15,500 mm × 13,200 mm × 4100 mm is established external to the electromagnetic exposure scenario. The governing equations are solved using COMSOL Multiphysics, a simulation platform based on the finite-element method (FEM), under predefined initial and boundary conditions. A high-quality mesh is generated for the entire computational domain; the element counts were 108,329 for the human torso, 10,473 for brain tissue, 22,674 for cardiac tissue, and 4066 for liver tissue. Figure 11 presents the mesh distribution of the complete model along with the configured boundary conditions.

### 2.4. Numerical Computation

#### 2.4.1. Electromagnetic Wave Propagation Analysis

The propagation of electromagnetic waves emitted by the RF antenna is governed by Maxwell’s equations, which describe the fundamental interdependence between electric and magnetic fields [38]. The governing equations are given in Equations (2)–(5).

(2) $$\nabla \times H=J+\frac{\partial D}{\partial t}$$

(3) $$\nabla \times E=-\frac{\partial B}{\partial t}$$

(4) ∇·B=0

(5) ∇·D=ρ

In these equations, ***H*** denotes the magnetic field intensity (A/m), ***J*** denotes the current density (A/m^2^), ***D*** denotes the electric flux density (C/m^2^), ***E*** denotes the electric field intensity (V/m), ***B*** denotes the magnetic flux density (T), and ρ denotes the free charge density (C/m^3^).

To characterize the electromagnetic dose distribution within the human body model, Maxwell’s equations can be reformulated into the wave equation shown in Equation (6).

(6) $$\nabla \times (\frac{1}{\mu_{r}}\nabla \times E)-k_{0}^{2}(\varepsilon_{r}-\frac{j\sigma }{\omega \varepsilon_{0}})E=0$$

where $\mu_{r}$ is the relative permeability, *n* is the refractive index, $\varepsilon_{r}$ is the relative permittivity, $\varepsilon_{0}$ = 8.8542 × 10^−12^ F/m represents the permittivity of free space, σ is the electrical conductivity (S/m), *j* is the imaginary unit, and $k_{0}$ is the free-space wave number (m^−1^).

In order to avoid reflections of the emitted electromagnetic waves, a cuboidal domain was constructed around the entire model. The scattering boundary condition was applied to the outer surfaces of this cuboidal domain, as expressed below [19]:

(7) $$n\times (\nabla \times E)-\mathrm{jkn}\times (E\times n)=-n\;\times \;(E_{0}\times \mathrm{jk}(n-k))\exp(-\mathrm{jk}\cdot r)$$

where *k* is the wave number (m^−1^), *n* is the normal vector, and *j* = $\sqrt{-1}$ and $E_{0}$ comprise the incident plane wave (V/m).

#### 2.4.2. Specific Absorption Rate (SAR)

When human tissues are exposed to RF electromagnetic fields, the incident waves radiated from the antenna interact with biological media, and part of the electromagnetic energy is absorbed by the tissues. Because the absorbed electromagnetic dose cannot be directly measured in vivo, numerical dosimetry has become a widely adopted method for quantifying electromagnetic energy absorption in biological tissues. The internationally accepted metric for absorbed RF energy is the SAR [39,40], defined as the electromagnetic power absorbed per unit mass of tissue. SAR is calculated using Equation (8).

(8) $$\mathrm{SAR}=\frac{\sigma {\left|E\right|}^{2}}{2\rho }$$

where *σ* is the electrical conductivity of the tissue (S/m), ***E*** is the induced electric field within the biological tissue (V/m), and ρ is the tissue density (kg/m^3^).

#### 2.4.3. Bioheat Transfer Theory

Biological effects induced by electromagnetic waves include both thermal and non-thermal mechanisms. Non-thermal effects refer to interactions between electromagnetic fields and biomolecules that alter physiological processes without causing significant temperature changes. Thermal effects arise when electromagnetic energy absorbed by biological tissues is converted into heat, resulting in a temperature rise. Temperature rise in tissues exposed to RF radiation can be described using the Pennes bioheat transfer equation [41,42,43,44]. First proposed by Harry H. Pennes in 1948 while analyzing the relationship between resting forearm tissue temperature and arterial blood temperature, the Pennes model extends the classical heat conduction equation to incorporate blood perfusion. The bioheat equation is shown in Equation (9).

(9) $$\rho C\frac{\partial T}{\partial t}=\nabla \cdot (k\nabla T)+\rho_{b}C_{b}\omega_{b}(T_{b}-T)+Q_{\mathrm{met}}+Q_{\mathrm{ext}}$$

where ρ is the tissue density (kg/m^3^), *C* is the specific heat capacity (J/(kg·°C)), *k* is the thermal conductivity (W/(m·°C)), *T* is the tissue temperature (°C), $T_{b}$ is the blood temperature (°C), $\rho_{b}$ is the blood density (kg/m^3^), $C_{b}$ the blood specific heat capacity (J/(kg·°C)), $\omega_{b}$ is the blood perfusion rate (s^−1^), $Q_{\mathrm{met}}$ is the metabolic heat generation (W/m^3^), and $Q_{\mathrm{ext}}$ is the external heat source (W/m^3^).

During thermal analysis, heat exchange between tissue and blood is approximated by the perfusion term $\rho_{b}C_{b}\omega_{b}(T_{b}-T)$. The total heat source in the model comprises metabolic heat $Q_{\mathrm{met}}$ and heat dissipation produced by electromagnetic absorption $Q_{\mathrm{ext}}$, as shown in Equation (10).

(10) $$Q_{\mathrm{ext}}=\frac{1}{2}\sigma_{\mathrm{tissue}}{\left|E\right|}^{2}=\rho \cdot \mathrm{SAR}$$

where $\sigma_{tissue}$= $2\pi f{\varepsilon_{r}^{\prime }\varepsilon }_{0}$, with $\varepsilon_{r}^{\prime }$ denoting the real part of tissue relative permittivity and $\varepsilon_{0}$ the permittivity of free space.

This paper assumes no contact resistance exists between human internal organs, with their internal boundaries set as continuous ones [36]:

(11) $$n\cdot (k_{u}\nabla T_{u}-k_{d}\;\nabla \;T_{d})=0$$

The surface of the human body is assumed to be a convective boundary condition:

(12) $$-n\cdot (-k\nabla T)=h_{b}(T-T_{i})$$

where $h_{b}$ is the convection coefficient of blood (W/$m^{2}\cdot K$), and $T_{i}$ is the ambient temperature (°C).

Because electromagnetic waves also undergo energy loss while propagating in free space, the initial tissue temperature is set to 310.15 K (37 °C). To simplify the computation, the following assumptions are made:

1. Electromagnetic waves directly interact with the human body and are completely absorbed by the tissues.
2. Interactions between electromagnetic waves and tissues occur in an open-field environment.
3. A scattering boundary condition is applied to truncate the free-space region surrounding the body.
4. Thermal properties of all tissues are assumed to be homogeneous and constant.

In this paper, a coupled multiphysics numerical model is established by combining the bioheat transfer equation with Maxwell’s equations. Numerical simulations are performed to analyze the temperature rise in the tissues of the subway station attendant caused by the absorption of RF electromagnetic energy in a typical subway station environment.

#### 2.4.4. Numerical Calculation Method

Bioelectromagnetic dosimetry consists of two major branches: experimental dosimetry and numerical dosimetry. Experimental dosimetry refers to the direct measurement of energy deposition within biological organisms or physical models under controlled electromagnetic exposure. Numerical dosimetry, in contrast, involves solving Maxwell’s equations for a given exposure scenario through computational modeling and simulation [45]. Because the electromagnetic dose absorbed by human tissues cannot be directly measured in vivo, numerical dosimetry has become an indispensable approach for quantifying electromagnetic energy deposition in biological tissues. This paper utilizes the COMSOL simulation software to model and solve the electromagnetic–bioheat-transfer thermal-coupling process based on the FEM, in order to assess the occupational exposure safety of the subway station attendant.

ICNIRP published exposure guidelines in 1998, 2010, and 2020 covering electromagnetic fields from 0 Hz to 300 GHz [8,46,47]. These guidelines establish scientifically based exposure limits aimed at protecting both the general public and occupational workers. The basic restrictions specified in ICNIRP (2020) [8] are listed in Table 3. With respect to tissue temperature, ICNIRP (2020) [8] stipulates that the allowable temperature increase in human tissues should not exceed 1 °C.

### 2.5. Verification of the Model Validity

To ensure the accuracy of the proposed methodology and the reliability of the obtained results, a numerical validation was performed prior to investigating the electromagnetic radiation effects of ceiling-mounted antennas on subway station attendants. Accordingly, a benchmark model consistent with that reported in [48] was established using the COMSOL Multiphysics platform. The validation was conducted by comparing the simulated SAR and temperature rise with the reference data.

**Figure 12:**
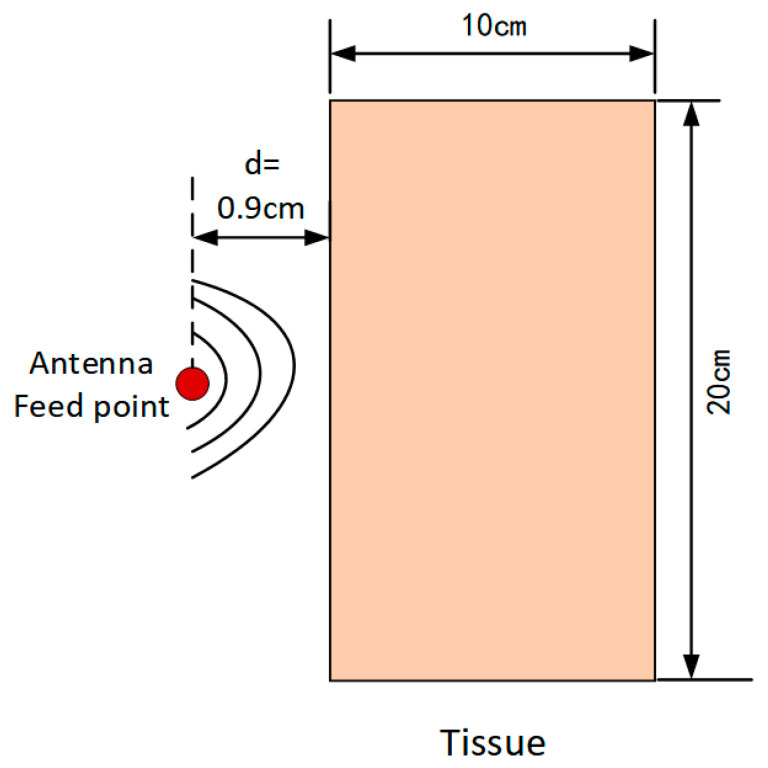
Validation of the computational model.

**Figure 13:**
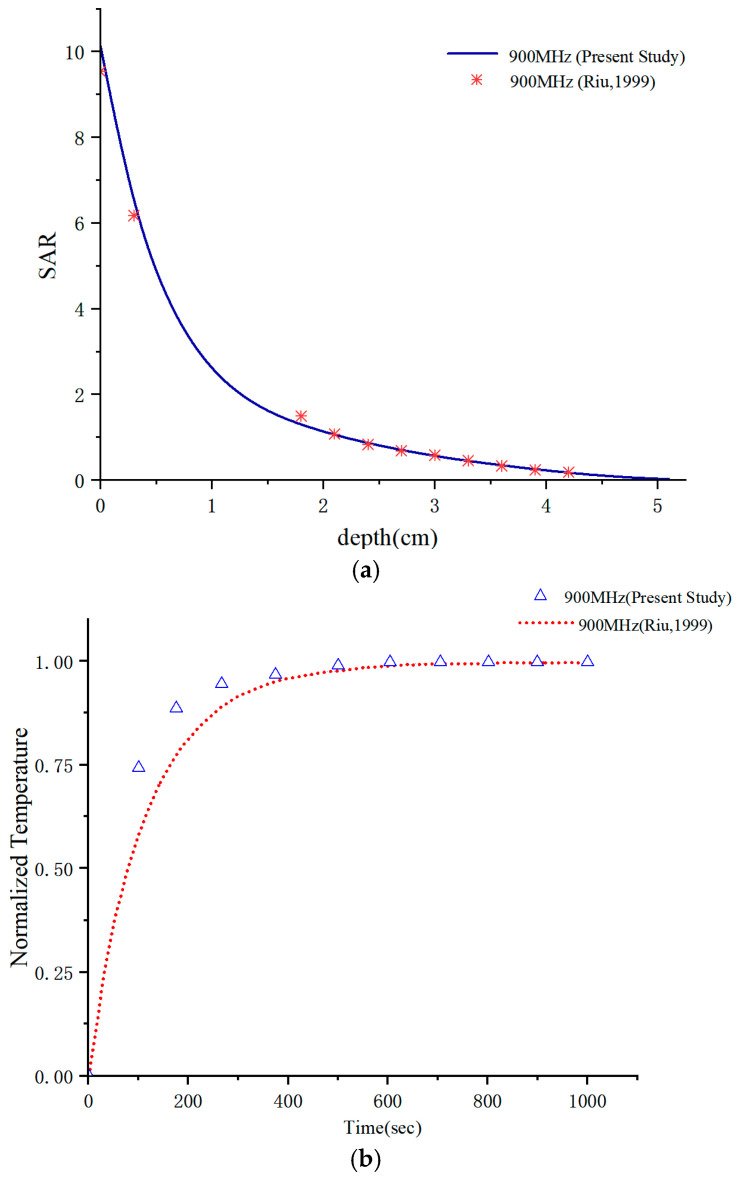
The calculated results were compared with those of Riu: (**a**) SAR, (**b**) normalized temperature [48].

As illustrated in Figure 12, a homogeneous tissue model exposed to the near-field radiation of a dipole antenna was constructed. As shown in Figure 13a, the simulated SAR and temperature rise exhibit excellent agreement with the results reported by [48]. Furthermore, Figure 13b indicates that the steady-state temperature rise obtained in Reference [48] is 0.045, while the corresponding value calculated in this study is 0.04357, resulting in a relative error of 3.18%. This close agreement effectively validates the accuracy and reliability of the proposed numerical modeling approach.

## 3. Analysis of the Calculation Results

### 3.1. SAR Distribution

**Figure 14:**
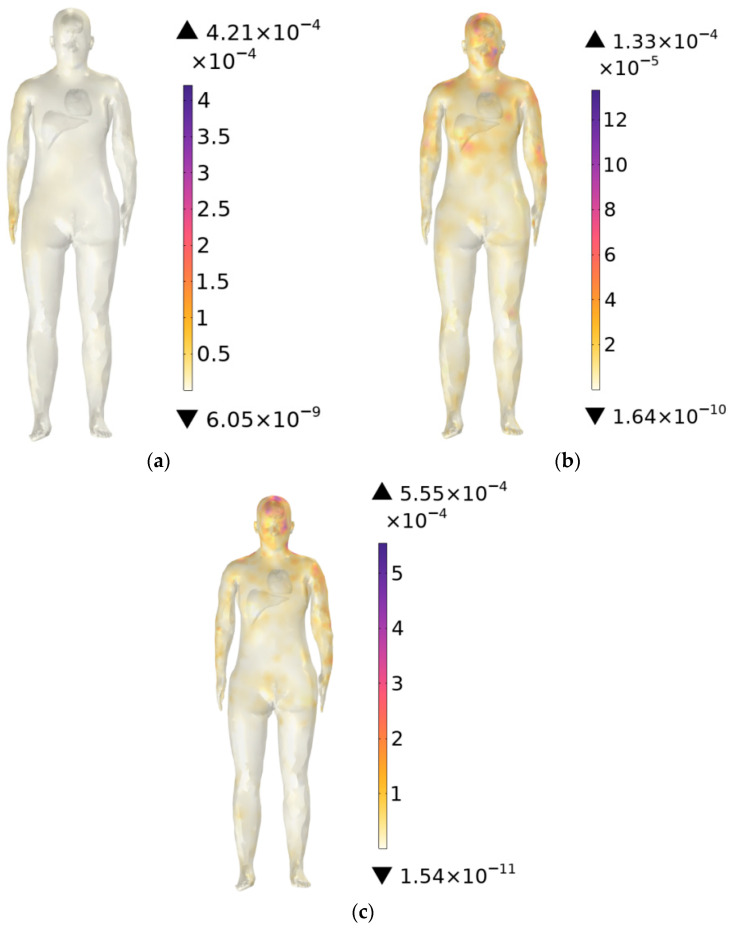
SAR of the trunk: (**a**) 900 MHz, (**b**) 2600 MHz, and (**c**) 3500 MHz.

Figure 14a–c show the SAR distribution in the trunk at operating frequencies of 900 MHz, 2600 MHz, and 3500 MHz. The results indicate that the SAR distribution varies markedly with frequency. At 900 MHz, the maximum SAR appears in the arm region closest to the antenna. At 2600 MHz, the highest SAR is concentrated in the head, while at 3500 MHz, the maximum values occur in both the head and neck regions. Across all three frequencies, the highest SAR in the trunk is observed at 3500 MHz, with a value of 5.55 × $10^{-4}$ W/kg. The maximum SAR values at all frequencies remain well below the ICNIRP occupational exposure limit of 0.4 W/kg.

Figure 14a–c show SAR maxima shift from the arm (900 MHz) to the head (2600–3500 MHz). This is because with increasing frequency, the wavelength decreases and the penetration depth reduces. Low-frequency bands have longer wavelengths with energy concentrated in the arm, whereas high-frequency bands have shorter wavelengths, shallower penetration depth, and energy mainly concentrated in the superficial tissue layers.

To investigate the effect of different antenna powers on electromagnetic exposure levels, this study calculated the SAR at antenna powers of 1 W and 10 W, respectively. The calculation results are 8.42 × $10^{-3}$ W/kg and 0.0842 W/kg, respectively. Simulation results show that SAR values have a positive linear correlation with antenna powers, consistent with the physical principle that electromagnetic energy absorbed by biological tissues is proportional to radiated power.

### 3.2. Temperature Distribution

#### 3.2.1. Temperature Rise in Human Tissues at 900 MHz

Considering that environmental temperatures in subway stations typically range from 18 °C to 28 °C (18 °C in winter, 28 °C during summer or peak periods, and 25 °C under normal operating conditions), the ambient temperature is set to 25 °C, with an initial body temperature of 37 °C. Temperature distributions in the trunk (excluding the brain, heart, and liver) are computed at exposure durations of 1 min, 6 min, and 30 min at 900 MHz.

**Figure 15:**
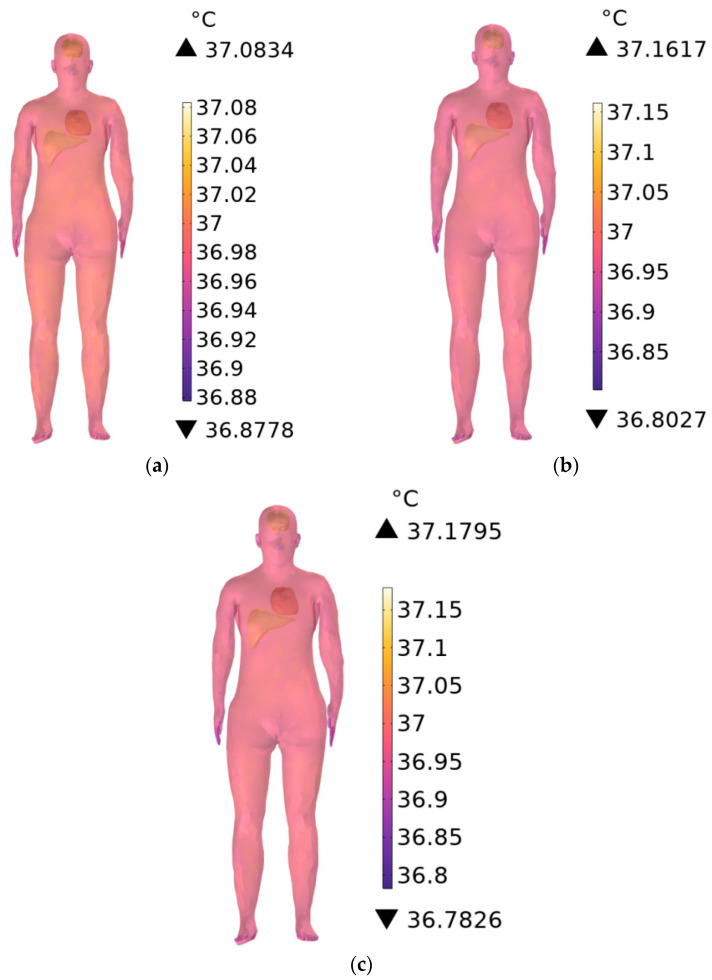
Temperature distribution of the trunk when the exposure time is (**a**) 1 min, (**b**) 6 min, and (**c**) 30 min.

Figure 15a–c show the temperature distribution of the station attendant’s trunk when the RF antennas operate at 900 MHz and the exposure durations of 1 min, 6 min, and 30 min, respectively. Compared with the initial body temperature, the trunk temperature increases by 0.0834 °C, 0.1617 °C, and 0.1795 °C, respectively, with longer exposure durations yielding higher temperature rises. The minimum trunk temperatures at 1 min, 6 min, and 30 min are 36.8778 °C, 36.8027 °C, and 36.7826 °C, respectively; these values are slightly below the initial 37 °C, due to convective heat transfer at the interface between the skin and the environment, most prominently near the fingertips.

**Figure 16:**
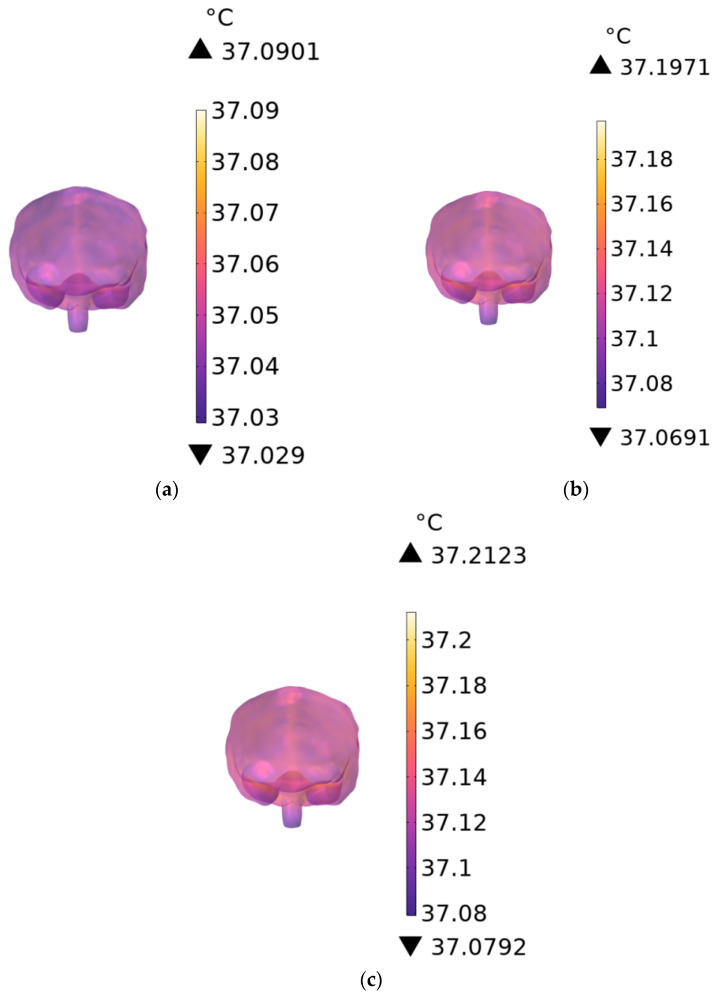
Temperature distribution of the brain when the exposure time is (**a**) 1 min, (**b**) 6 min, and (**c**) 30 min.

Figure 16a–c show the temperature rise in brain tissue when the RF antennas operate at 900 MHz. At exposure durations of 1 min, 6 min, and 30 min, the temperature increases by 0.0901 °C, 0.1971 °C, and 0.2123 °C, respectively. The observed thermal increase is slightly greater than that of the trunk due to lower cerebral blood perfusion and reduced heat dissipation.

**Figure 17:**
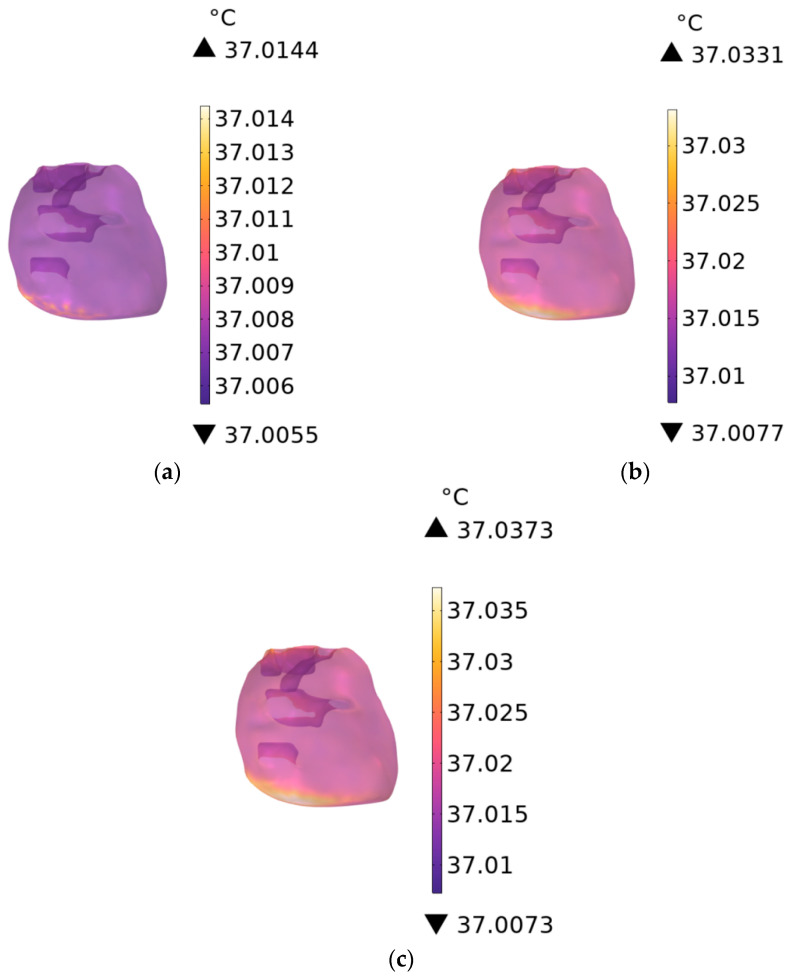
Temperature distribution of the heart when the exposure time is (**a**) 1 min, (**b**) 6 min, and (**c**) 30 min.

**Figure 18:**
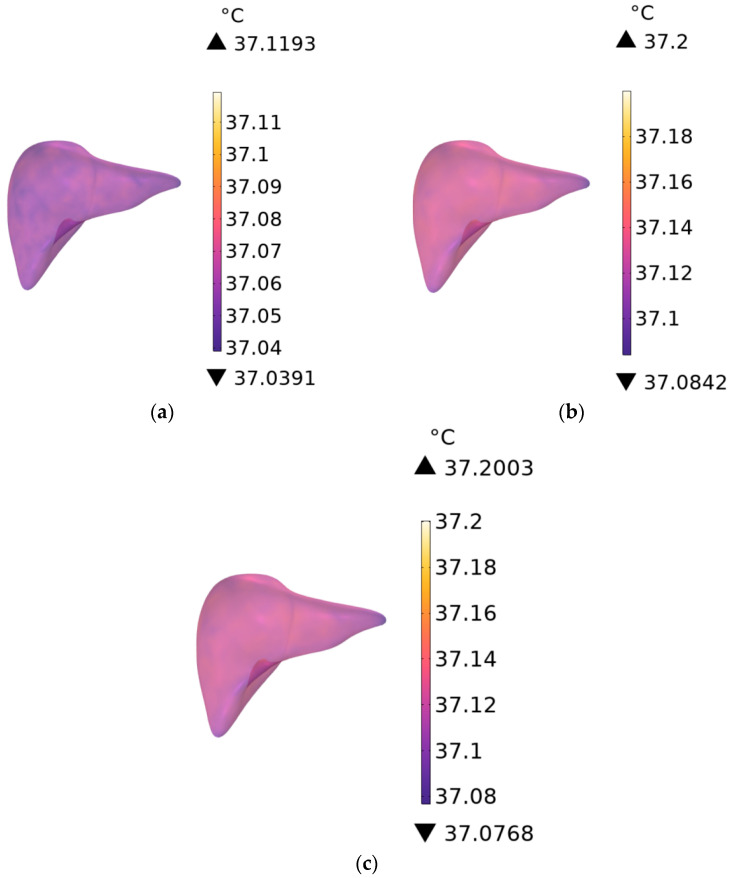
Temperature distribution of the liver when the exposure time is (**a**) 1 min, (**b**) 6 min, and (**c**) 30 min.

As shown in Figure 17a–c, the temperature distribution of human heart tissue is presented for RF antennas operating at 900 MHz with exposure durations of 1 min, 6 min, and 30 min, respectively, and that of liver tissue in Figure 18a–c under the same conditions. The temperature rise in the heart tissue is 0.0144 °C, 0.0331 °C, and 0.0373 °C, respectively, and that in the liver tissue is 0.1193 °C, 0.2000 °C, and 0.2003 °C, respectively. 

#### 3.2.2. Temperature Rise Curves at 900 MHz

**Figure 19:**
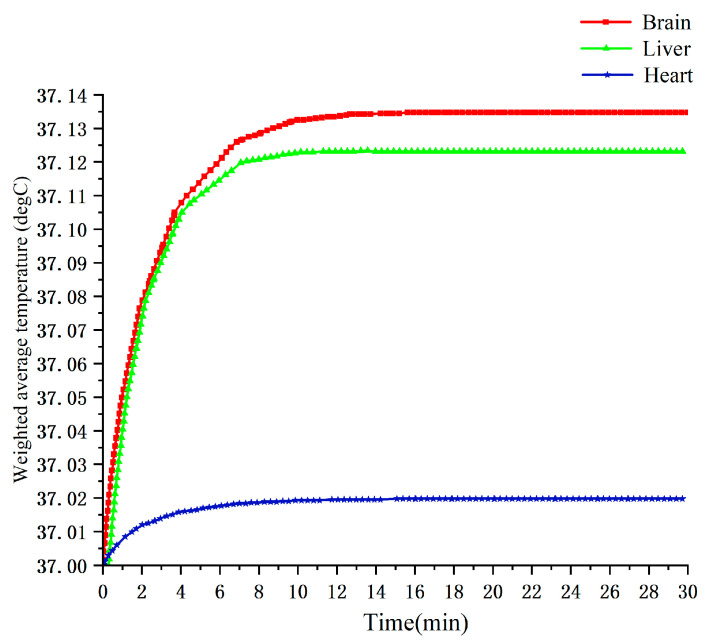
Temperature change curves of brain, heart, and liver tissues from 0 to 30 min at 900 MHz.

Figure 19 illustrates the temperature evolution of the brain, heart, and liver over 0–30 min of exposure at 900 MHz with an ambient temperature of 25 °C. The three tissues exhibit clearly distinguishable thermal responses: the brain shows the largest temperature rise, while the heart shows the smallest. This is primarily due to differences in blood perfusion. The brain has relatively low perfusion and thus dissipates heat inefficiently, leading to faster accumulation; the heart, with higher perfusion, dissipates heat more effectively, resulting in a slower temperature increase [19].

#### 3.2.3. Temperature Rise Under Different Frequencies and Ambient Conditions

**Figure 20:**
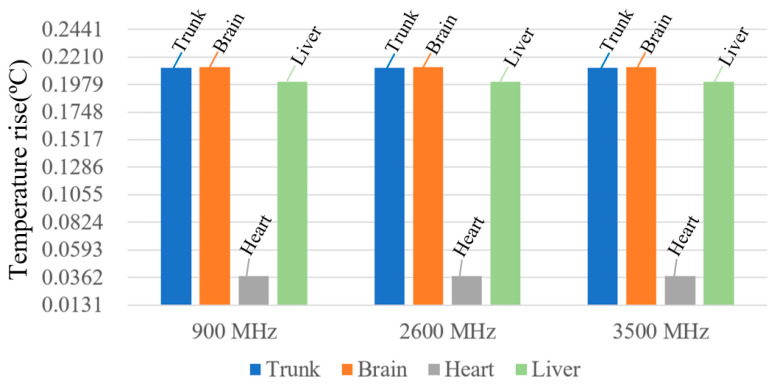
Maximum temperature rise in human tissue at different frequencies.

With the ambient temperature set to 25 °C (non-peak operational periods), the maximum temperature rise in human tissues at operating frequencies of 900 MHz, 2600 MHz, and 3500 MHz is shown in Figure 20. At these three frequencies, the maximum temperature rise in the station attendant’s trunk is 0.21207333 °C, 0.21207313 °C, and 0.21207311 °C, respectively. The variation in peak tissue temperature across frequencies is minimal. This is because the large distance between the human body and the radiation source results in an extremely small baseline temperature rise; the frequency-independent thermophysical properties of tissues—thermal conductivity, specific heat capacity, and blood perfusion rate—fundamentally limit the magnitude of temperature variation. Meanwhile, the inherent thermal conduction and diffusion effects of tissues can rapidly offset local temperature differences, ultimately leading to minimal differences in the overall temperature rise across different frequencies.

**Figure 21:**
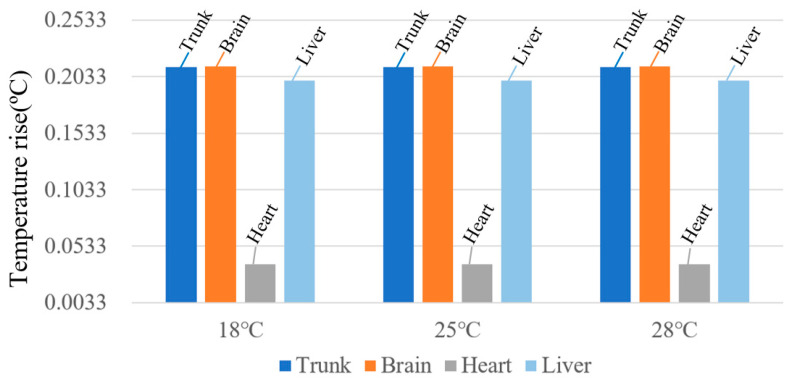
Steady-state temperature rise in human tissues at different ambient temperatures.

When the RF antenna operates at 900 MHz, the temperature rise in various human tissues is calculated under ambient temperatures of 18 °C, 25 °C, and 28 °C, as shown in Figure 21. The results indicate that the steady-state temperature rise in human tissues is essentially the same at different ambient temperatures. This behavior arises from the body’s robust thermoregulatory capacity when the ambient temperature is well below the core temperature of 37 °C. Under RF exposure, heat dissipation through blood perfusion effectively mitigates the influence of elevated ambient temperature, resulting in only minor variations in tissue heating. These findings suggest that the thermal response remains relatively stable within typical environmental temperature ranges.

Overall, after 30 min of exposure, all tissues reach a steady state. The highest temperature rise is observed in brain tissue at 900 MHz under the 18 °C winter ambient condition, with a maximum increase of 0.2123 °C, corresponding to 21.23% of the ICNIRP temperature-rise limit.

#### 3.2.4. The Impact of the Built Environment on Human Body Temperature Rise

**Figure 22:**
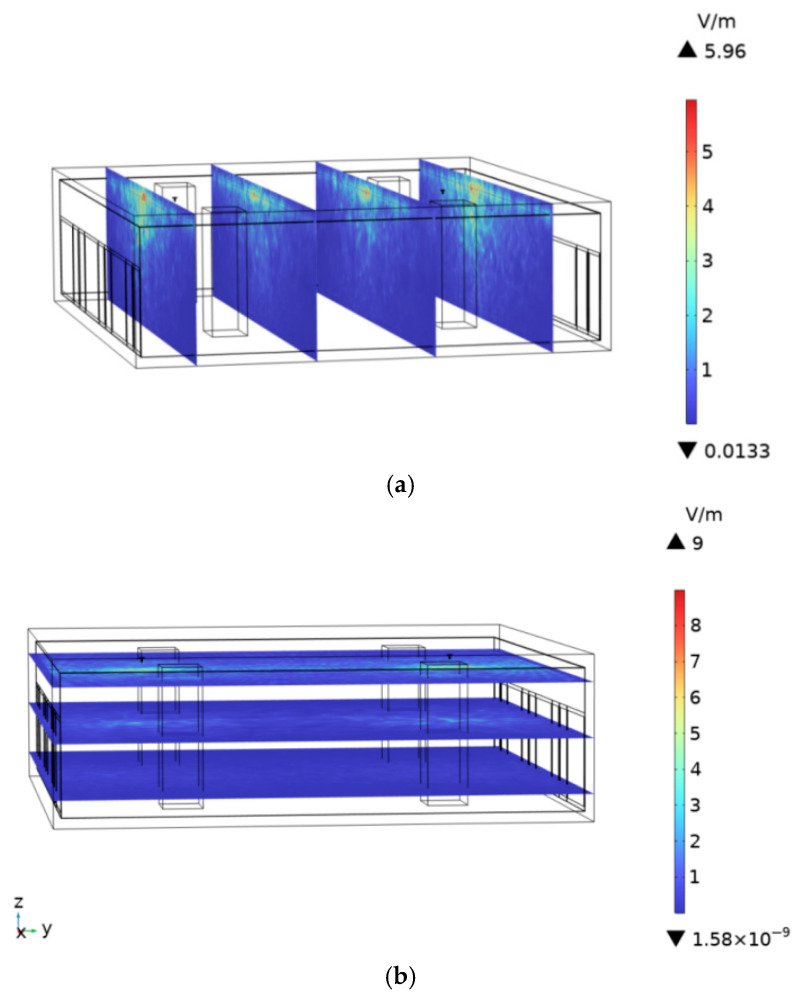
Electric field intensity distribution in the presence of subway station buildings: (**a**) XZ plane, (**b**) XY plane.

**Figure 23:**
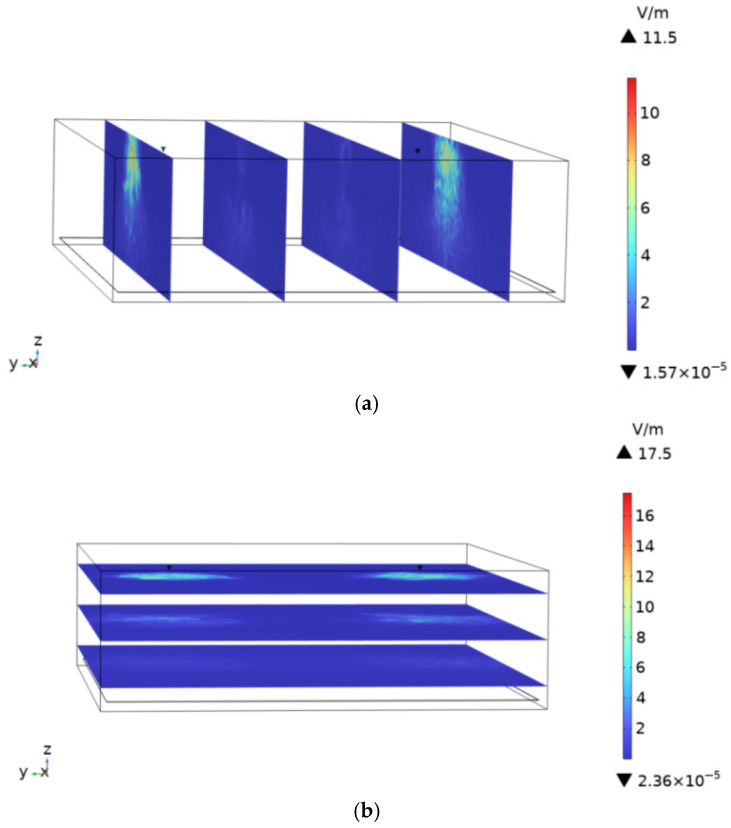
Electric field intensity distribution in the absence of subway station buildings: (**a**) XZ plane, (**b**) XY plane.

To assess the impact of the built environment on human body temperature rise, the spatial electric-field distribution and corresponding tissue heating are compared between scenarios with and without a subway station environment. At an ambient temperature of 25 °C and the antenna operating frequency of 900 MHz, the spatial electric-field distribution in the subway station environment is shown in Figure 22a,b. The maximum field strengths are 5.96 V/m in the XZ plane and 9.0 V/m in the XY plane. In the computational model, the ceiling, concrete columns, and platform screen doors of the subway station are removed, and the resulting spatial electric-field distribution is shown in Figure 23a,b. In the absence of these structural elements, the maximum electric-field strengths reached 11.5 V/m in the XZ plane and 17.5 V/m in the XY plane. This increase arises because the built environment introduces reflection and partial electromagnetic shielding or absorption. Consequently, compared with an open environment without a built environment, the presence of station infrastructure reduces the spatial electric-field intensity within the subway station.

**Figure 24:**
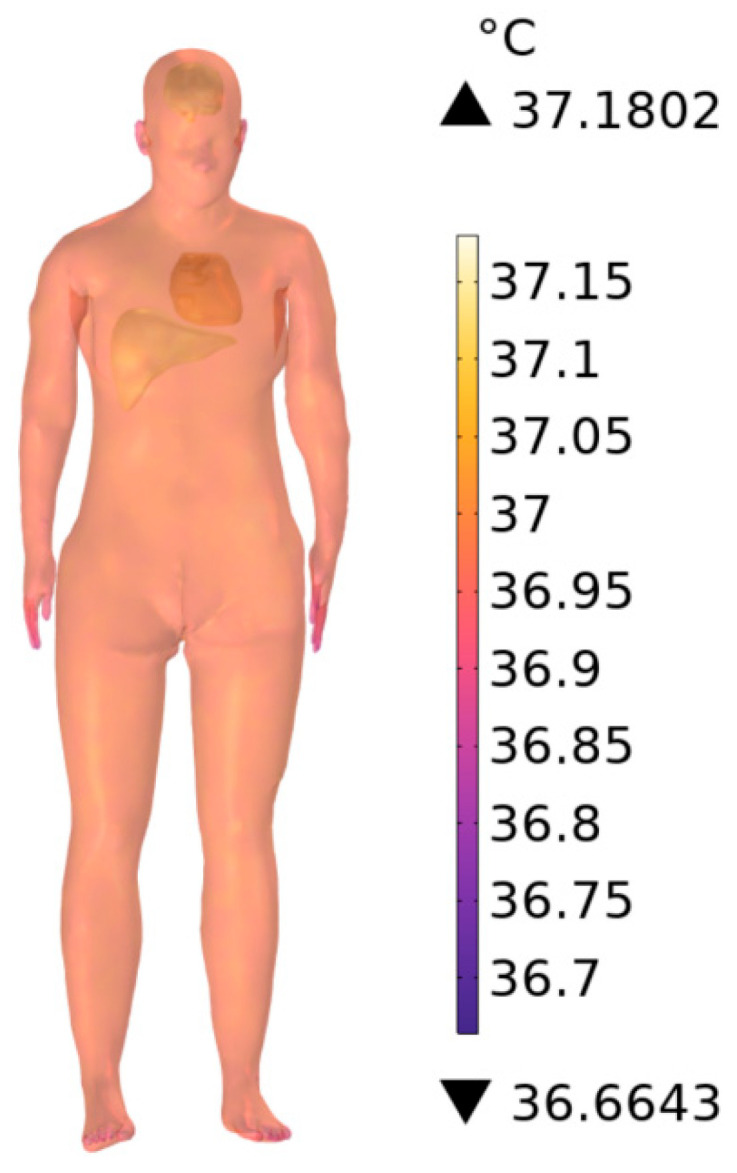
Steady-state temperature rise in the human body at 900 MHz in the absence of subway station buildings.

Figure 24 shows the steady-state tissue temperature rise in the absence of subway station buildings scenario at 900 MHz, yielding a maximum increase of 0.1802 °C. Compared with the 0.1795 °C observed in the scenario with subway station buildings, the temperature rise increases by 0.39%, confirming that ambient field strength directly influences tissue heating, though the magnitude of this effect remains small under far-field exposure conditions.

#### 3.2.5. The Effects of Different Positions on Human Electromagnetic Exposure

**Figure 25:**
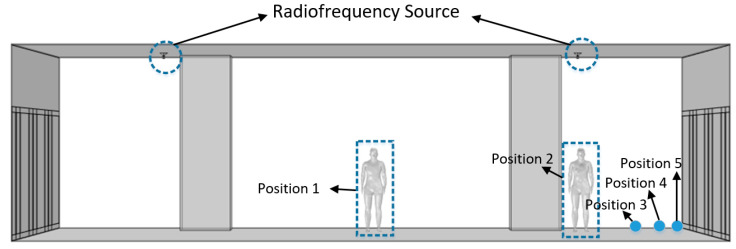
Model diagram of subway station attendants at different positions.

To evaluate the electromagnetic exposure level of station attendants at different work positions in a subway station, the present study established five simulation models, with the operating frequency of the antenna set at 900 MHz. The station attendants were located at five specific positions as illustrated in Figure 25. Position 1 was directly below the midpoint between the two antennas, at a distance of 6.4 m from the platform screen door. Position 2 was directly below one of the antennas, at a distance of 2.4 m from the platform screen door. Positions 3, 4, and 5 were at distances of 1 m, 0.5 m, and 0.3 m from the platform screen door, respectively. The simulation results of the five positions are presented in Table 4.

Table 4 shows that the whole-body SAR of the station attendant reached a relatively high value of 4.71 × $10^{-4}$ W/kg at Position 2, and the maximum value of 5.10 × $10^{-4}$ W/kg appeared at Position 3. This is because the vertical polarization characteristic of the antenna results in a higher electric field intensity at the lower side than that directly below the antenna. With the increase in the distance from one antenna, the SAR decreased at Position 4. Positions 2, 3, and 4 were of the same order of magnitude. Position 5 was far from the antenna and close to the platform screen door, which partially shielded and absorbed the electromagnetic waves, leading to the minimum SAR of 8.45 × 10^−5^ W/kg. Position 1 was subjected to the superposition of radiation from the two antennas, but due to the long distance from the antennas, the SAR achieved the second minimum value of 9.44 × 10^−5^ W/kg.

The results shown in Table 4 indicate that the maximum temperature rise in the station attendant also occurred at Position 3, with a peak value of 0.1863 °C, while the minimum temperature rise of 0.1701 °C was observed at Position 5. The variation trend of temperature rise at different positions was consistent with that of SAR, but the overall fluctuation range was relatively small. The SAR at Position 3 was 6.04 times that at Position 5, whereas the temperature rise at Position 3 was only 1.095 times that at Position 5. This is because the absorption of electromagnetic energy is not the only factor causing temperature change; instead, it is also affected by heat dissipation mechanisms, such as the heat conduction of human tissues themselves, resulting in a small difference in temperature rise among various positions [19].

## 4. Discussion

In this paper, we developed an RF antenna model representative of subway station environments and constructed a high-fidelity human model of a station attendant, incorporating critical organs such as the brain, heart, and liver. A realistic electromagnetic exposure scenario was also built, including architectural elements, such as the ceiling, concrete columns, and platform screen doors. Considering the typical temperature range within subway stations (18–28 °C), we evaluated the spatial distributions of SAR and temperature rise in human tissues under seasonal conditions for RF antenna operation at 900 MHz, 2600 MHz, and 3500 MHz. The main findings are summarized as follows:

(1) In this study, the SAR distribution within the body of subway station attendants varied significantly with operating frequency. At 900 MHz, the SAR peak occurred in the arm closest to the antenna; at 2600 MHz, it was located in the head; at 3500 MHz, it was observed in the head and neck regions. The maximum SAR values in References [10,21] also occurred in the trunk and head.
(2) Temperature rise in key organs and tissues was computed for RF antenna exposure at different frequencies, with irradiation durations of 1 min, 6 min, and 30 min. The results show that when the exposure duration reaches 30 min, the temperature rise in all critical tissues attained a steady-state condition. Owing to the combined influence of thermal conductivity, dielectric properties, and blood perfusion rates, the RF-induced temperature increase in biological tissues was not directly proportional to the SAR distribution. The maximum temperature rise occurred in brain tissue under winter station conditions with an ambient temperature of 18 °C at 900 MHz, reaching 0.2123 °C, which corresponded to 21.23% of the 1 °C temperature-rise limit specified by the ICNIRP guidelines.
(3) At an operating frequency of 900 MHz, a comparative analysis was performed between scenarios with and without surrounding architecture structures in a subway station environment. In the scenario without ceilings, platform screen doors, and concrete columns, the maximum spatial electric field intensity reached 17.5 V/m, exceeding the 11.5 V/m observed with buildings. The maximum tissue temperature rise in the scenario without architecture structures was 0.1802 °C, slightly higher than the 0.1795 °C recorded with structural elements present, and both values remain well below the ICNIRP guideline limit of 1 °C. These findings indicate that architectural structures exert reflective, partially absorptive, and shielding effects on electromagnetic wave propagation.

Despite providing reliable numerical insights into the electromagnetic exposure of subway station attendants, this study had notable limitations: the exclusion of passenger factors (no multi-body coexistence scenario analysis) and the adoption of static exposure analysis with a single posture and fixed position, which neglects the dynamic movements and posture changes of attendants in real work settings.

To mitigate these limitations, future research will focus on four key aspects:

(1) Developing a multi-body coupled model and investigating the effect of passenger density variations in different time periods on exposure levels;
(2) Developing a dynamic exposure assessment model considering the movement of subway station attendants;
(3) Investigating the electromagnetic exposure of subway station attendants in other work areas, such as station entrances;
(4) This study is limited to the female digital human model with a specific height of 1.63 m (Ella), and the results of studies on different statures may vary.

## 5. Conclusions

In summary, this study investigated the electromagnetic exposure characteristics of female subway station attendants of similar physical characteristics to the Ella model under the action of RF antennas. All calculation results indicate that their SAR and tissue temperature rise are both below the limit requirements of the ICNIRP, which demonstrates that the radiation emitted by RF antennas in subway stations poses a low health risk to attendants. These results can provide support for the electromagnetic environment assessment of subway stations; this data also lays a scientific foundation for formulating electromagnetic exposure protection strategies for subway staff and provides important evidence for the development of relevant electromagnetic exposure standards for rail transit systems. However, considering that station attendants are subject to long-term continuous exposure to this electromagnetic environment in their daily work, the potential long-term health impacts still require sustained attention.

## Figures and Tables

**Table 1:** Dielectric parameters of different human tissues.

Tissue	900 MHz		2600 MHz		3500 MHz	
	$\varepsilon_{r}$	**σ (S/m)**	$\varepsilon_{r}$	**σ (S/m)**	$\varepsilon_{r}$	**σ (S/m)**
Brain	45.8055	0.76653	42.33	1.6033	41.154	2.223
Heart	59.893	1.2298	54.508	2.3795	52.831	3.2048
Liver	46.833	0.85497	42.79	1.7879	41.417	2.4677
Trunk	42.56275	0.8727225	39.924755	1.619515	38.94075	2.1909675

**Table 2:** Thermal parameters of different human tissues.

Human Tissue	ρ ($\mathrm{kg}/m^{3}$)	*k* (W/(m·°C))	*C* (J/(kg·°C))	$\omega_{b}$ ($s^{-1}$)
Brain	1038	0.53	3650	0.00883
Heart	1050	0.43	3638	0.02
Liver	1079	0.52	3540	0.01505
Trunk	1293.5	0.4375	2987.75	0.0051837

**Table 3:** Basic restrictions for electromagnetic field exposure from 100 kHz to 6 GHz, for averaging intervals ≥ 6 min.

Exposure Scenario	Frequency Range	Whole-Body Average SAR (W/kg)
Occupational	100 kHz–6 GHz	0.4

**Table 4:** Calculation results at different positions.

	Position 1	Position 2	Position 3	Position 4	Position 5
SAR(W/kg)	$9.44\;\times \;10^{-5}$	$4.71\;\times \;10^{-4}$	$5.10\;\times \;10^{-4}$	$4.21\;\times \;10^{-4}$	$8.45\;\times \;10^{-5}$
Temperature rise(°C)	0.1791	0.1798	0.1863	0.1795	0.1701

## Data Availability

The original contributions presented in this study are included in the article. Further inquiries can be directed to the corresponding author.
